# Parenting Young Children: The Interplay Between Mothers’ and Fathers’ Daily Behaviors and Well-Being

**DOI:** 10.3390/bs16020230

**Published:** 2026-02-05

**Authors:** Dorit Aram, Linor Sagi, Hadar Hazan

**Affiliations:** 1 School of Education, Tel Aviv University, Tel Aviv 6997801, Israel; 2 Department of Psychology, College of Health Sciences, State University of New York Polytechnic Institute, Utica, NY 13502, USA; hazanh@sunypoly.edu

**Keywords:** dyadic parenting, parental well-being, general well-being, beneficial parenting behaviors, Parenting Pentagon Model, partner effects

## Abstract

This dyadic study distinguishes parents’ general well-being (overall life satisfaction) from parental well-being (satisfaction specific to the parenting role) and examines how each relates to daily beneficial parenting behaviors in mother–father couples. Guided by the Parenting Pentagon Model (PPM), five behavioral constructs—Partnership, Leadership, Expressions of Love, Encouraging Independence, and Adherence to Rules—were assessed in 170 Israeli parents (85 mother–father dyads) of children aged 6 months to 9 years. Parents reported frequent beneficial parenting, with Expressions of Love the most prevalent and Encouraging Independence and Adherence to Rules the least frequent. Mothers reported significantly higher Expressions of Love than fathers (*p* < 0.01), with no gender differences for the other PPM constructs. Across both parents, higher engagement in beneficial parenting behaviors was consistently associated with higher levels of both general and parental well-being (actor effects), with stronger associations for mothers than fathers. Partner effects showed a clear gender asymmetry: fathers’ parenting behaviors were positively associated with mothers’ general and parental well-being, whereas mothers’ behaviors were not consistently associated with fathers’ well-being. In addition, a larger number of children was negatively associated with mothers’ parental well-being. Overall, the findings highlight the relevance of daily parenting behaviors for parents’ own well-being and underscore the relational nature of parenting, with fathers’ behaviors playing a particularly salient role in mothers’ well-being within families of young children.

## 1. Introduction

Parenting is a dynamic process that fosters growth in both children and parents. However, much empirical literature examines isolated parenting behaviors or single-parent perspectives (e.g., maternal anger or persistence), rather than parenting as coordinated, dyadic daily practices within the family system (Nomaguchi & Milkie, 2020). Research on beneficial parenting behaviors, characterized by sensitivity, affection, and balanced discipline, consistently links them to positive child adjustment across cultures (Baumrind, 1967). Less attention has examined how these behaviors relate to parents’ own well-being (Le et al., 2018) or spillover effects between partners (Kouros et al., 2014; Nomaguchi et al., 2015). Thus, a central question remains: Are parents’ daily beneficial parenting behaviors associated with their own general well-being (overall life satisfaction) and parental well-being (role-specific satisfaction), as well as their partner’s well-being, within mother-father dyads?

Israel provides a theoretically relevant context, for examining these dyadic parenting processes. Prior research characterizes Israeli parents as highly involved in early childhood, emphasizing emotional closeness and family interdependence (Sotzker-Cohen, 2002). Importantly, Israel has a relatively high proportion of two-parent families (World Population Review, 2026) and birth rate (Kubovich, 2023), creating a family context where daily parenting practices occur within active coparenting partnerships. Combined with patterns of maternal employment and paternal involvement (Aram et al., 2022; Chen et al., 2014), this setting is ideal for examining cross-partner well-being associations and spillover processes, contributing to cross-cultural PPM literature (Aram et al., 2022; Meoded Karabanov et al., 2021). Accordingly, this study examined how mothers’ and fathers’ PPM behavioral constructs relate to their own and partners’ general and parental well-being. We tested actor (within-parent) and partner (cross-partner) effects and whether these differed by parent gender.

The Parenting Pentagon Model (PPM) (Aram et al., 2022) conceptualizes parenting as five observable daily behavioral constructs: Partnership between caregivers, Leadership, Expressions of Love, Encouraging Independence, and Adherence to Rules. Grounded in family systems theory (Cox & Paley, 1997), the PPM views parenting as an interdependent process where parental behaviors and well-being emerge through reciprocal relational dynamics within the family system.

Baumrind’s foundational work (Baumrind, 1966) emphasized balancing responsiveness and control, a principle consistently linked to positive child and family outcomes across cultures and developmental stages (Cipriano & Stifter, 2010; Gorman-Smith et al., 2000; Mandara et al., 2012). Building on this authoritative framework, the PPM reconceptualizes parenting as coordinated dyadic behaviors, articulated through its five constructs that capture everyday family functioning (Alon et al., 2025; Meoded Karabanov et al., 2021).

The PPM has been validated in cross-cultural studies (Aram et al., 2022; Meoded Karabanov et al., 2021; Meoded Karabanov et al., 2025), supporting its relevance across sociocultural contexts. These five constructs define the core elements of beneficial daily parenting behaviors.

**Partnership** involves collaboration and communication between caregivers, encompassing mutual support, shared responsibilities, and consistent involvement. High partnership links to children’s independence, self-regulation, and social skills (Newland et al., 2008; Ashton-James et al., 2013; Bellon et al., 2017).

**Leadership** encompasses parents establishing family values, organizing activities, monitoring progress, decision-making, and role modeling. Effective leadership fosters children’s autonomy and achievement (Yu et al., 2015; Yuliani et al., 2019; Perra et al., 2021).

**Expressions of Love** include affection, sensitivity, empathy, quality time, and minimal criticism, strengthening attachment and promoting positive emotions and cooperation (Nelson et al., 2014; Shin et al., 2023; Carlson, 2023).

**Encouraging Independence** fosters autonomy through age-appropriate independence support, enhancing executive function and prosocial behaviors (Guay et al., 2018; Laurin & Joussemet, 2017; LeCuyer, 2014).

**Adherence to Rules** establishes consistent household norms, linking to children’s health and normative development (Mueller et al., 2024; Roskam et al., 2014; Montesino et al., 2021).

### 1.1. Links to Parents’ Well-Being

General well-being refers to individuals’ self-evaluations of happiness and life satisfaction (Buecker et al., 2023). A higher level of general well-being is linked to fewer mental health symptoms, stronger relationships, and better functional health (Mathentamo et al., 2024; Suldo & Shaffer, 2008). Parenting behaviors are closely tied to parents’ happiness and sense of meaning (Nomaguchi & Milkie, 2020); however, much existing literature emphasizes sociodemographic as predictors of well-being (e.g., age, income, work hours) and focuses predominantly on mothers (Bellon et al., 2017; Luhmann et al., 2012), with fathers’ involvement and well-being receiving comparatively less attention (McKeown et al., 2003). Research indicates that dimensions such as general life satisfaction, satisfaction with the parenting role, and marital satisfaction are interconnected but conceptually distinct (McKeown et al., 2003). This study addresses this gap by examining associations between PPM behavioral constructs and both parents’ general and parental well-being, within individuals and across partners.

Parental well-being refers to the subjective well-being reflected in the parenting experience. Researchers evaluate parental well-being through parents’ self-reports of ‘positive’ emotions, such as joy and a sense of meaning, and ‘negative’ emotions, such as sadness, stress, and fatigue (Aassve et al., 2012; Bellon et al., 2017; Musick et al., 2016; Voydanoff & Donnelly, 1998). Since parents make decisions that affect all family members, parental well-being plays a significant role in family dynamics and overall well-being, which, in turn, are associated with children’s well-being and resilience (Nelson et al., 2014).

### 1.2. Parental Everyday Behavior and Well-Being

Studies of parenting behaviors and well-being have typically examined comparative or situational analyses, including differences between parents and non-parents (Ballas & Dorling, 2007), well-being shifts during parenthood transition (Impett et al., 2011), and parents’ well-being during child time versus other activities (Offer, 2014). Far less research examines everyday parenting behaviors as predictors of parents’ own well-being.

Addressing this gap, the present study examines parental well-being through the lens of daily parenting behaviors, focusing on how mothers’ and fathers’ everyday practices are associated with both their own and their partner’s well-being within the parental dyad. Evidence supports positive associations between specific everyday parenting behaviors and parental well-being, including parental competence and effectiveness (Leadership) (Fiese et al., 2005), parent–child shared time (Expressions of Love) (McKeown et al., 2003), encouragement of independence (Edlund & Öun, 2023), and consistent family routines (Adherence to Rules) (Faircloth & Murray, 2015).

Since parenting behaviors are enacted within a gendered family context, sociodemographic factors, particularly parents’ gender, are central to understanding how daily parenting behaviors relate to both mothers’ and fathers’ well-being.

### 1.3. Socio-Demographic Measures Related to Parenting and Parents’ Well-Being

**Gender**. Parental gender significantly influences the relationship between parenting behaviors and well-being (Faircloth & Murray, 2015). In the present study, we examined how mothers’ and fathers’ parenting behaviors relate to their respective general and parental well-being. Although researchers indicate increased paternal involvement, gender disparities in housework and childcare responsibilities persist across much of the Western world (Lee, 2023; Craig & Mullan, 2011). The rise in women’s employment has not substantially reduced their caregiving burden, as many mothers continue to shoulder the majority of family care responsibilities (Sayer & Gornick, 2012; Rinaldi & Howe, 2012).

Regarding well-being, fatherhood is often associated with positive outcomes, as fathers report higher life satisfaction and happiness and lower depression rates than childless men (Bellon et al., 2017). Findings regarding mothers are more complex: some studies indicate higher well-being among mothers compared to non-mothers (Nomaguchi & Milkie, 2020); others report no differences or even lower well-being among mothers (Orchard et al., 2022; McMahon et al., 2015).

**Age**. The link between parental age and well-being remains unresolved. Some studies show that younger first-time parents experience more depressive symptoms (McMahon et al., 2015; Mirowsky & Ross, 2002). However, contrasting findings suggest no association between maternal age and depressive symptoms in the first two years postpartum (Stutzer & Frey, 2006).

**Number of children**. Research on family size and well-being yields mixed results. Some studies report that life satisfaction decreases with more children (Pollmann-Schult, 2018), while others find that the first child increases life satisfaction, but additional children do not (Augustine & Negraia, 2024).

**Parents’ education**. Higher parental education levels affect well-being in nuanced ways. Highly educated parents often derive fewer subjective benefits from parenting and report higher levels of stress and fatigue than less educated parents (Augustine & Negraia, 2024).

Together, these findings on gendered caregiving norms and sociodemographic factors suggest that mothers’ and fathers’ daily parenting behaviors may relate differently to their own and their partners’ well-being, providing a theoretical basis for our hypotheses.

### 1.4. Research Aims and Hypotheses

The present study examined daily parenting behavioral constructs (PPM) and their associations with general and parental well-being, addressing three aims:

1. Describe and compare PPM behavioral constructs (Partnership, Leadership, Expressions of Love, Encouraging Independence, Adherence to Rules) and levels of general and parental well-being between mothers and fathers.
2. Examine within-parent associations between each parent’s PPM behavioral constructs and their own general and parental well-being, controlling for sociodemographic factors (age, education, and family size).
3. Investigate cross-partner associations between parents’ PPM behavioral constructs and their partner’s general and parental well-being.

**H1.**  *(PPM behavioral constructs). Mothers and fathers will exhibit higher Expressions of Love than Adherence to Rules and Encouraging Independence. The five PPM constructs will positively correlate within parents and between partners within families.*

**H2.**  *(Well-being levels). Fathers will report higher general and parental well-being than mothers.*

**H3.**  *(Associations). Beneficial PPM behavioral constructs will positively correlate with parents’ own well-being, controlling for socio-demographics. Fathers’ PPM behavioral constructs will show stronger positive associations with mothers’ well-being than vice versa.*

## 2. Materials and Methods

### 2.1. Participants

The participants included 170 Israeli parents (85 couples—mothers and fathers). The sample largely represented Jewish parents with young children in Israel, excluding the Ultra-Orthodox population. Families had two to four children, with 54.10% having two, 38.8% having three, and 7.1% having four.

The children’s age range was six months to nine years. The parents’ ages ranged from 25 to 47 years (*M* = 35.46, *SD* = 4.61) for the mothers and 30 to 54 years for the fathers (*M* = 38.17, *SD* = 5.08). Mothers’ education varied: 21.20% had secondary education, 10.60% held a professional diploma, 28.20% had a bachelor’s degree (BA), 37.60% had a master’s degree (MA), and 2.4% held a Ph.D. In the Israeli context, maternal education is widely used as a reliable proxy for family socioeconomic status (SES), particularly in administrative and population-based research where detailed household income data are often unavailable. Maternal education reflects integration into the labor market, access to economic resources, and broader social mobility processes, including immigrant background effects (Akiva et al., 2024). Fathers’ education levels were as follows: 24.7% had secondary education, 12.9% held a professional diploma, 36.5% had a BA, 20.2% had an MA, and 4.7% held a Ph.D. Average weekly working hours were 33.43 (*SD* = 14.50) for mothers and 46.83 (*SD* = 7.47) for fathers.

### 2.2. Measurements

#### 2.2.1. Parental Behavioral Constructs Questionnaire

Each parent completed a self-report questionnaire consisting of 74 items assessing daily parenting behaviors across the five constructs of the Parenting Pentagon Model (PPM). The questionnaire was developed through a systematic process of item generation grounded in theory and prior literature (Aram et al., 2022), followed by pilot testing and psychometric evaluation, as detailed below. The full questionnaire is available from the corresponding author upon request. Parents were asked to report the frequency of each behavior during a typical week on a 6-point Likert scale ranging from 1 (never) to 6 (always). Items were organized according to the five PPM constructs:

**Partnership**. Fourteen items described parents’ daily collaboration behaviors, such as “I support my partner in their reactions to our child” or “My partner and I discuss issues relating to our parenting.”

**Leadership**. Eighteen items described daily behaviors emphasizing parents’ roles as family leaders who organize family life and model desired behaviors (e.g., “I behave according to goals I have for raising my child”, “I plan my parenting behaviors; “I prepare for toilet training, weaning off a pacifier, and transitioning from preschool—I gather information, seek advice, etc.”).

**Expressions of Love**. Eighteen items described daily physical (e.g., “I hug my child”) and verbal expressions of love, sensitivity, and empathy toward the child (e.g., “I do small actions that will make my child happy, such as preparing food that he/she loves or buying small things”).

**Encouraging Independence**. Ten items described encouraging the child to perform age-appropriate tasks independently (e.g., “When my child asks me for help, I first suggest that he/she tries on his/her own” or “I encourage my child to be independent in daily activities such as dressing, showering, eating, or brushing teeth”).

**Adherence to Rules**. Fourteen items described parents’ daily adherence to household rules and routines (e.g., “When my child does not follow the rules, I make sure that there is a consequence” or “I remind my child of the rules of the house”).

The average score across each construct’s items constituted the construct score, with higher scores indicating more beneficial parenting behaviors. The questionnaire was developed by two child development researchers, three couples, and a family therapist and drew upon established measures of parenting (e.g., coparenting, parental warmth, positive parenting, and parental authority). Reliability was first examined in a pilot study with 40 couples (*n* = 20 mothers), demonstrating good internal consistency (Partnership *α* = 0.87; Leadership *α* = 0.87; Expressions of Love *α* = 0.87; Encouraging Independence *α* = 0.77; Adherence to Rules *α* = 0.83).

Confirmatory factor analysis (CFA), conducted using Mplus (Version 8.0), supported the questionnaire’s overall factor structure, with all items loading positively onto their intended constructs (loadings > 0.30). One item from the Encouraging Independence construct was removed following the pilot study, resulting in the final 10-item scale used in the present study.

In the current sample, internal consistency was high: Partnership (*α* = 0.93 for mothers; *α* = 0.87 for fathers), Leadership (*α* = 0.88 for mothers; *α* = 0.83 for fathers), Expressions of Love (α = 0.89 for mothers; *α* = 0.88 for fathers), Encouraging Independence (*α* = 0.77 for mothers; α = 0.78 for fathers), and Adherence to Rules (*α* = 0.84 for mothers; *α* = 0.82 for fathers).

A shorter version of the PPM questionnaire has been used with parents from diverse cultural contexts, including American, Bulgarian, Israeli Jewish, Israeli Arab, and Spanish populations. The PPM questionnaire and its psychometric properties have been published and applied in multiple peer-reviewed international journal articles and book chapters (Aram et al., 2022; Meoded Karabanov et al., 2021; Montesino et al., 2021).

#### 2.2.2. General Well-Being Questionnaire

General well-being was assessed using the Hebrew version of the Mental Health Inventory (MHI; Florian & Drori, 1990). This instrument includes 21 items designed to assess parents’ perceptions of their well-being over the past month, focusing on positive feelings about life and negative feelings such as sadness.

Parents rated each item on a 6-point Likert scale ranging from 1 (never) to 6 (always). Negative items were reverse-scored so that higher scores indicated fewer negative feelings. The average score across all items was calculated to represent overall general well-being.

The questionnaire demonstrated high internal consistency, with positive affect reliabilities of *α* = 0.91 for mothers and *α* = 0.89 for fathers, and negative affect reliabilities of *α* = 0.90 for mothers and *α* = 0.83 for fathers. The General Well-Being Questionnaire was introduced after completion of the pilot phase. As a result, data on general well-being were available for a subsample of 65 couples, whereas parental well-being and parenting behavior measures were available for the full sample of 85 couples.

#### 2.2.3. Parental Well-Being Questionnaire

Parents completed a self-report questionnaire consisting of 21 items assessing parental well-being, including positive feelings toward parenting (e.g., “I feel joy from my parenting”; “My children give me satisfaction and pleasure”) and negative feelings related to parenting (e.g., “I am frustrated with my role as a mother/father”; “I feel overwhelmed by the demands of being a parent”).

Parents rated the frequency of each experience on a 6-point Likert scale ranging from 1 (never) to 6 (always). Negative items were reverse-scored such that higher scores reflected fewer negative parenting-related emotions, and higher overall scores indicated higher parental well-being.

The questionnaire was developed specifically for the present study, drawing conceptually and empirically on established measures of parental well-being and stress, including the Parenting Stress Index (Abidin, 2012), the Parental Stress Scale (Berry & Jones, 1995), the Subjective Psychological Well-Being Indexes (Bryant & Veroff, 1982), the BBC Well-Being Scale (Kinderman et al., 2011), the Parenting Satisfaction Index (Libby et al., 2008), the Parental Feelings and Control Measure (Oliver et al., 2014), and the Child-Raising Interview (Perez & Fox, n.d.).

Reliability was initially established in a pilot study with 40 parents, demonstrating excellent internal consistency (Cronbach’s *α* = 0.94). In the current sample, internal consistency remained high for both mothers and fathers. Cronbach’s *α* coefficients for positive emotional items were 0.95 for mothers and 0.92 for fathers, while coefficients for negative emotional items were 0.89 for mothers and 0.86 for fathers.

#### 2.2.4. Demographic Questionnaire

The demographic questionnaire included 14 items capturing key family-related information, including the number of children, parents’ ages, and each parent’s level of education.

### 2.3. Procedure

The Ethics Committee of Tel Aviv University approved the study protocol. Participants were recruited using a snowball sampling approach that extended beyond the researchers’ personal networks. Questionnaires were disseminated through multiple institutional and community channels, including a national daycare network, kindergartens, and graduate counseling programs.

Data collection took place in participants’ homes. Each parent completed the questionnaires individually, without consulting the other parent. To ensure anonymity, no identifying information was collected. Completed questionnaires were sealed in envelopes and opened only at the university.

The study included 85 heterosexual couples, of whom 20 participated in the pilot study. Based on pilot findings, two additions were made to the final questionnaire: a demographic item regarding weekly working hours and the General Well-Being Questionnaire. Consequently, these data were available for only 65 couples.

### 2.4. Data Analysis

Assumptions of normality, linearity, homoscedasticity, and sphericity were evaluated using descriptive statistics, visual inspection of distributions/residual plots, and Greenhouse–Geisser corrections where violated. Multicollinearity was assessed using intercorrelations and variance inflation factors (VIFs). Descriptive statistics were computed for mothers’ and fathers’ parenting behaviors and well-being, with paired-sample t-tests comparing parents across measures. Two-way repeated-measures ANOVAs examined the PPM construct as the within-subject factor separately for mothers and fathers.

Pearson correlations examined associations among PPM parenting behaviors, well-being measures, and family background variables. To account for the dyadic structure, actor–partner interdependence models (APIM; Kenny et al., 2006) examined associations between parents’ parenting behaviors and both their own (actor effects) and their partner’s (partner effects) general/parental well-being. Wald χ^2^ tests compared corresponding actor and partner effects across parents.

## 3. Results

Section 3 reports on mothers’ and fathers’ daily parenting behaviors as defined by the PPM, their general and parental well-being, and within- and cross-parent associations between parenting and well-being.

### 3.1. Mothers’ and Fathers’ Daily Parenting Behaviors

Table 1 presents descriptive statistics for mothers’ and fathers’ reports of daily parenting behaviors across the five PPM constructs, along with between-parent comparisons. Overall, both mothers and fathers reported relatively high engagement in beneficial parenting behaviors. The highest mean scores were observed for Expressions of Love (mothers: *M* = 4.86, *SD* = 0.57; fathers: *M* = 4.70, *SD* = 0.57), indicating frequent expressions of affection, sensitivity, and shared time with the child.

Paired-sample *t*-tests revealed a significant gender difference only for the Love construct, with mothers reporting higher levels than fathers. No significant gender differences were found for Partnership, Leadership, Encouraging Independence, or Adherence to Rules. Moderate correlations were observed between mothers’ and fathers’ reports across all PPM constructs (*r* = 0.27 to 0.65), indicating concordance in parenting behaviors within couples.

To examine within-parent differences across the five PPM constructs, repeated-measures analyses of variance (ANOVA) were conducted separately for mothers and fathers. The analyses revealed a significant main effect of construct for mothers, *F* (4, 336) = 27.65, *p* < 0.001, η^2^ = 0.25, and for fathers, *F* (4, 336) = 25.65, *p* < 0.001, η^2^ = 0.23, indicating a hierarchical pattern among the PPM constructs.

**Figure 1:**
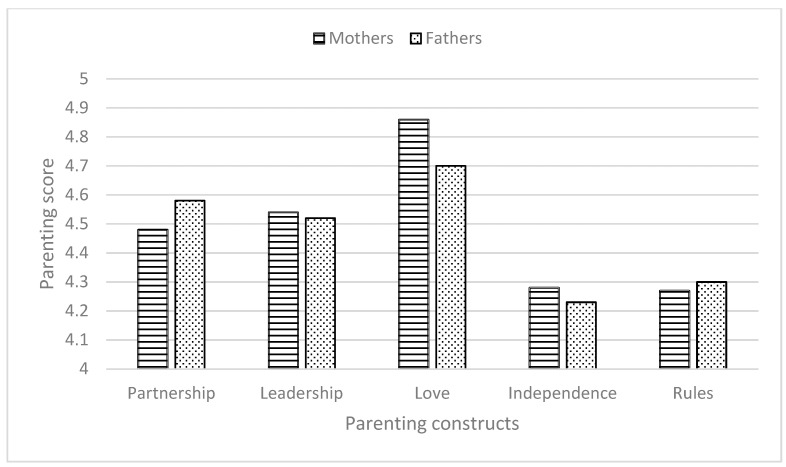
Description of Parents’ Reports of Their Implementation of the Five PPM Constructs (*N* = 85).

Bonferroni-corrected post hoc comparisons indicated that, for mothers, Love was reported significantly higher than all other constructs. Leadership did not differ significantly from Partnership but was significantly higher than Encouraging Independence and Adherence to Rules. A comparable pattern was observed among fathers: Love was reported to be significantly higher than Leadership, Encouraging Independence, and Adherence to Rules. Leadership did not differ significantly from Partnership but was significantly higher than Encouraging Independence and Adherence to Rules. Partnership did not differ significantly from Love or Leadership but was significantly higher than Encouraging Independence and Adherence to Rules (see Figure 1).

### 3.2. Mothers’ and Fathers’ General and Parental Well-Being

Both mothers and fathers reported relatively high levels of general and parental well-being, with positive feelings reported more frequently than negative feelings (see Table 1). Paired-sample t-tests revealed that mothers reported significantly higher levels of negative feelings related to general well-being (*t* = 3.07, *p* < 0.01) and negative feelings related to parenting (*t* = 2.32, *p* < 0.05) compared to fathers.

Significant correlations were observed between mothers’ and fathers’ positive and negative feelings toward parenting: fathers’ greater positive feelings were associated with mothers’ greater positive feelings, and fathers’ greater negative feelings were associated with mothers’ greater negative feelings. A similar pattern emerged for general well-being within couples, but only for positive feelings (see Table 1). To assess potential bias related to the availability of general well-being data, we compared couples who completed the general well-being questionnaire (*N* = 65) with couples from the pilot study who did not complete it (*N* = 20) on key demographic variables and parenting measures available for the full sample. No significant differences were found between the groups (all *p*s > 0.10).

We then created integrated general well-being (*α* = 0.86 for mothers; *α* = 0.87 for fathers) and parental well-being (*α* = 0.95 for mothers; *α* = 0.93 for fathers) variables. Before analyzing the impact of parenting behaviors (PPM) on well-being, we conducted Pearson correlation analyses between family background measures (number of children in the family, fathers’ and mothers’ ages, and education) and mothers’ and fathers’ general and parental well-being. The only significant association indicated that the number of children in the family was negatively correlated with mothers’ parental well-being (*r* = −0.29, *p* < 0.01), such that mothers with more children reported lower parental well-being.

### 3.3. Associations Between Parenting Behavioral Constructs (PPM) and General and Parental Well-Being: Actor–Partner Interdependence Models

In this section, we examined how each of the five PPM constructs (Partnership, Leadership, Love, Encouraging Independence, and Adherence to Rules) was associated with mothers’ and fathers’ general and parental well-being using actor–partner interdependence models (APIM). These models simultaneously estimated actor effects (associations between a parent’s own parenting behaviors and their own well-being) and partner effects (associations between one parent’s behaviors and the other parent’s well-being). For descriptive purposes, zero-order correlations between parenting behaviors and parental and general well-being are reported in Appendix A. The full set of APIM estimates and model fit indices is presented in Table 2 (general well-being) and Table 3 (parental well-being).

#### 3.3.1. General Well-Being

General well-being APIM analyses were conducted on the subsample of 65 mother–father dyads. With regard to general well-being, among mothers, all actor estimates were relatively similar, indicating that higher scores on leadership, partnership, and love were associated with higher general well-being (Partnership: *β* = 0.72, *p* < 0.001; Leadership: *β* = 0.63, *p* < 0.001; Love: *β* = 0.62, *p* < 0.001), whereas actor effects for Encouraging Independence and Adherence to Rules were somewhat smaller (*β* = 0.23, *p* < 0.05; *β* = 0.40, *p* < 0.001, respectively). Among fathers, all actor estimates were significant, yet lower than those of mothers across all five constructs (Partnership: *β* = 0.46, *p* < 0.01; Leadership: *β* = 0.46, *p* < 0.01; Love: *β* = 0.46, *p* < 0.001; Encouraging independence: *β* = 0.25, *p* < 0.05; Adherence to Rules: *β* = 0.26, *p* < 0.05).

Partner effects for general well-being were less consistent. Fathers’ parenting behaviors showed positive partner effects on mothers’ general well-being for Encouraging Independence and Adherence to Rules (*β* = 0.44, *p* < 0.001; *β* = 0.21, *p* < 0.05, respectively), indicating that higher paternal engagement in these behaviors co-occurred with higher maternal general well-being. In contrast, no significant partner effects were observed from mothers’ parenting behaviors to fathers’ general well-being.

We further compared model estimates to test whether the actor effects of the five PPM constructs differed. These comparisons indicated that actor effects did not differ significantly across constructs, suggesting that the associations between the different parenting behaviors and general well-being were similar in magnitude. In an additional step, we compared actor effects across parents, focusing on whether the association between Leadership and general well-being differed for mothers and fathers. A Wald χ^2^ test indicated a significant difference in the Leadership effect on general well-being (χ^2^ = 4.04, *df* = 1, *p* = 0.04), whereas no other parent differences were observed.

Because no significant partner effects were observed from mothers’ parenting behaviors to fathers’ general well-being, no further partner-effect comparisons were conducted. Model results indicated adequate explanatory power across the five general well-being models, as reflected by the variance explained (*R*^2^) and additional fit indices reported in Table 2.

#### 3.3.2. Parental Well-Being

Table 3 presents the results for parental well-being. Among mothers, significant positive actor effects of comparable magnitude were observed across all five PPM constructs (Partnership: *β* = 0.66, *p* < 0.001; Leadership: *β* = 0.64, *p* < 0.001; Love: *β* = 0.73, *p* < 0.001; Encouraging Independence: *β* = 0.29, *p* < 0.01; Adherence to Rules: *β* = 0.44, *p* < 0.001). Among fathers, all actor effects were likewise significant (Partnership: *β* = 0.50, *p* < 0.001; Leadership: *β* = 0.70, *p* < 0.001; Love: *β* = 0.74, *p* < 0.001; Encouraging Independence: *β* = 0.35, *p* < 0.001; Adherence to Rules: *β* = 0.32, *p* < 0.01).

Significant partner effects were also observed, such that fathers’ Leadership and Encouraging Independence were positively associated with mothers’ parental well-being (*β* = 0.17, *p* < 0.05; *β* = 0.26, *p* < 0.01, respectively). In contrast, no significant partner effects emerged from mothers’ PPM behaviors to fathers’ parental well-being, consistent with the pattern observed for general well-being.

**Figure 2:**
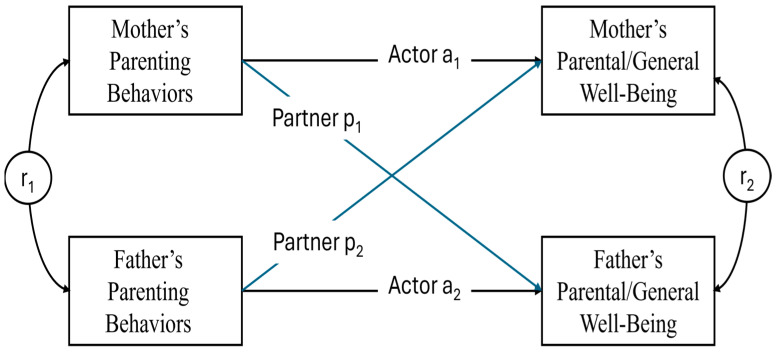
Dyadic Model of Parenting Behaviors and Well-Being. *Note.* Actor–Partner Interdependence Model (APIM) examining associations between Parenting Pentagon Model (PPM) constructs and parental well-being. Solid paths indicate significant actor effects (a1, a2); dashed paths indicate significant partner effects (p1, p2). Correlations between partners’ parenting behaviors are shown (r1, r2). Models adjust for number of children. Only significant paths are displayed.

The number of children was negatively associated with mothers’ parental well-being, with larger family size predicting lower well-being in the Leadership (*β* = −0.17, *p* < 0.01), Encouraging Independence (*β* = −0.33, *p* < 0.001), and Adherence to Rules (*β* = −0.31, *p* < 0.01) models. No corresponding associations were observed for fathers’ parental well-being. Post hoc Wald tests comparing actor and partner effects did not reveal significant differences among these estimates (See Figure 2).

## 4. Discussion

This study presents an integrated examination of parenting behaviors exhibited by both mothers and fathers of young children within families, viewed via the lens of the Parenting Pentagon Model (PPM). It examines how daily parenting behaviors (Partnership between the parents, Leadership, Love expression, Encouraging Independence, and Adherence to Rules) are associated with parents’ general and parental well-being, both within individuals and across partners, beyond key family background characteristics. The Discussion is organized around the study’s three aims: (1) to characterize beneficial parenting behaviors, (2) to examine their associations with parents’ well-being, and (3) to explore cross-gender associations within the parental dyad.

Guided by the study hypotheses, the findings largely supported expectations regarding both the distribution of PPM behavioral constructs and their associations with well-being. As hypothesized, mothers and fathers reported more frequent expressions of love than all other behaviors, as well as higher levels of partnership and leadership relative to encouragement of independence and Adherence to Rules, reflecting a hierarchical pattern of daily parenting practices during early childhood. Consistent with expectations, moderate-to-strong positive associations were observed between partners within couples across parenting behaviors, indicating substantial concordance in parenting practices. At the same time, subsequent dyadic analyses revealed that coordination in parenting behaviors does not necessarily translate into symmetrical influence processes, highlighting the importance of distinguishing between behavioral alignment and cross-parent effects on well-being.

The hypotheses concerning general and parental well-being were partially supported and refined by the present findings. Mothers and fathers reported similarly high levels of positive general and parental well-being; however, mothers reported significantly higher levels of negative affect than fathers in both domains. As hypothesized, beneficial parenting behaviors were positively associated with parents’ own general and parental well-being beyond relevant demographic factors, as reflected in robust actor effects for both mothers and fathers.

Importantly, the findings extended the original hypotheses by revealing asymmetric cross-parent associations. Fathers’ daily parenting practices showed significant partner effects on mothers’ general and parental well-being, whereas corresponding effects from mothers to fathers were not observed. This asymmetry points to gendered dynamics in spillover processes and underscores the importance of considering differentiated parental roles and influence pathways within the family system (Kouros et al., 2014; Repetti et al., 2015).

### 4.1. Parenting Behavioral Constructs and Gender Differences

Consistent with prior studies, both mothers and fathers reported frequent engagement in beneficial parenting practices across all five daily parenting behavior constructs. The prominence of Love behaviors toward young children is consistent across cultures, with Bulgaria, Spain, and the United States reporting more Expressions of Love behaviors than other constructs (Aram et al., 2022).

A significant gender difference was observed only in the Love construct, with mothers reporting higher expressions of love than fathers. This difference likely reflects mothers’ greater involvement in daily caregiving during early childhood, heightened emotional attunement, and societal norms that position caregiving and emotional responsiveness as central to maternal identity (Milkie et al., 2002; Rajhans et al., 2019). Neuroimaging studies further suggest that mothers of infants tend to exhibit greater activation in limbic regions associated with emotional processing, whereas fathers show stronger activation in social–cognitive brain networks (Lewis & Lamb, 2003). Although fathers’ involvement in childcare has increased, they may continue to conceptualize their role through indirect forms of support, such as contributing to family stability via leadership and partnership behaviors (Gervais et al., 2021).

No significant gender differences were found in the other PPM constructs (Partnership, Leadership, Encouraging Independence, and Rules), suggesting growing alignment in parenting practices within families. This finding is consistent with research documenting increased paternal engagement and shared caregiving roles in families with young children (Gervais et al., 2021; Hodkinson & Brooks, 2018; Sotzker-Cohen, 2002).

The relatively lower emphasis on Encouraging Independence and adherence to Rules aligns with prior research indicating that Israeli parents often adopt a flexible approach to rules, prioritizing emotional closeness over early emotional autonomy (Meoded Karabanov et al., 2021). Israeli parenting emphasizes high parental involvement, strong closeness, and child-centered daily life, which is frequently expressed as “doing a lot for the child” (Klimor-Maman et al., 2023; Scharf, 2013). Israeli parents tend to grant children considerable freedom in everyday interactions and impose relatively few behavioral restrictions (Chen et al., 2014). While such parenting practices may foster strong emotional bonds, they may also contribute to increased emotional dependency on parents, particularly mothers, who often shoulder a disproportionate share of emotional and organizational caregiving responsibilities.

The positive, significant correlations between mothers’ and fathers’ parenting practices across all PPM constructs underscore the interdependence of parenting within families. These findings are consistent with spillover processes, whereby one parent’s behaviors are associated with the other parent’s behaviors and emotional experiences (Kouros et al., 2014). Similarities in parenting practices may arise from shared values, reciprocal learning, and intentional co-regulation of parenting strategies (Repetti et al., 2015; Schofield et al., 2009).

### 4.2. Parents’ Well-Being (General and Parental) and Its Predictors

Both mothers and fathers reported relatively high levels of general and parental well-being, consistent with prior research documenting generally positive orientations toward parenting and life satisfaction among parents of young children (Skreden et al., 2012; Herbst & Ifcher, 2016). However, mothers reported significantly higher levels of negative emotions than fathers in both general and parental well-being. This pattern is consistent with previous findings indicating that mothers often experience greater role strain, particularly in the context of balancing employment and caregiving responsibilities (Nomaguchi & Milkie, 2020; Giallo et al., 2011), and tend to report lower subjective well-being compared to fathers (Nelson et al., 2014). Beyond caregiving demands, mothers frequently carry a disproportionate share of emotional labor and family organization, which may contribute to elevated stress and emotional fatigue.

Socio-demographic factors further contextualized these findings. Larger family size was negatively associated with maternal well-being, a pattern that aligns with prior research linking increased caregiving demands to reduced subjective well-being and fewer opportunities for rest and self-care (Nelson et al., 2014; Aassve et al., 2012; Pollmann-Schult, 2018). Notably, this association was observed for mothers but not for fathers, underscoring potential gender differences in how family structure relates to parental well-being.

Beneficial parenting practices were strongly associated with both general and parental well-being for mothers and fathers. These findings extend prior work that has primarily emphasized child outcomes by demonstrating that daily parenting behaviors are also closely linked to parents’ own emotional health and sense of competence. Engagement in Partnership, Leadership, Love, Encouraging Independence, and Adherence to Rules behaviors may be associated with heightened feelings of efficacy, coherence, and satisfaction within the family system, which, in turn, are linked to higher well-being (Bandura et al., 2011).

Importantly, the present findings indicate that parenting dimensions are not merely interchangeable indicators of a single underlying construct. Rather, the APIM analyses revealed that specific parenting practices exert independent and construct-specific associations with parental and general well-being, including cross-parent effects that are not fully captured by an integrated composite score. Notably, certain paternal practices demonstrated unique partner associations with mothers’ well-being, suggesting that some dimensions of parenting carry explanatory power beyond overall parenting quality. Thus, construct-level analyses are essential for capturing the autonomous contributions of distinct parenting dimensions and for clarifying how specific practices operate within interdependent family systems.

### 4.3. Cross-Gender Influences

The findings demonstrate cross-gender associations between parenting practices and their general and parental well-being, consistent with an interdependent family system (Repetti et al., 2015). Notably, fathers’ parenting practices, particularly Leadership behaviors, Encouraging Independence and Adherence to Rules, showed significant partner effects on mothers’ general and parental well-being, whereas no significant partner effects emerged from mothers’ practices on fathers’ well-being.

The observed gender asymmetry in spillover effects can be understood in light of research on gendered parental norms. As Hays (1998) articulated in her formulation of intensive mothering, cultural expectations position mothers as primarily responsible for children’s emotional, developmental, and organizational needs, frequently demanding sustained emotional investment and self-sacrifice. Although fathers’ involvement in childcare has increased, mothers remain disproportionately responsible for key aspects of parenting and tend to report lower well-being than fathers (Milkie et al., 2002; Nomaguchi & Milkie, 2020).

Within this context, fathers’ engagement in daily parenting practices may be especially salient for mothers’ well-being, as such involvement may be associated with lower perceived caregiving burden and signal greater equity in parental roles (Edlund & Öun, 2023; Craig & Mullan, 2011). Mothers’ parenting behaviors, while central to family functioning, may be more socially expected and therefore less strongly associated with fathers’ well-being. These findings suggest that spillover processes are embedded within broader gendered expectations surrounding parenting, which shape how mothers and fathers differentially experience and benefit from daily parenting practices.

This asymmetry highlights the potential role of fathers’ parenting practices in supporting mothers’ well-being. Specifically, mothers appear to receive a well-being boost from fathers’ adherence to Rules and Encouraging Independence constructs—areas in which both parents show relatively weaker endorsement. These practices may be particularly valuable for children, while mothers’ greater sensitivity to child behavior could explain why fathers’ contributions in these domains provide mothers with meaningful relief from caregiving demands. Fathers’ Leadership behaviors also ease the mothers’ parental load. Moreover, greater overall paternal involvement appears to be linked to higher maternal satisfaction (Milkie et al., 2002).

### 4.4. Practical Implications

These findings have important implications for parenting interventions and family counseling. By focusing on observable daily behaviors rather than abstract beliefs or attitudes, the PPM offers a practical framework for helping parents identify strengths and areas for growth in their parenting practices. Interventions that support concrete parenting behaviors across the five PPM dimensions may help reduce caregiving strain, enhance parental well-being, and promote healthier family dynamics. Importantly, the dyadic findings suggest that parenting practices operate within an interdependent family system, underscoring the potential value of engaging both parents in intervention efforts.

Given the cross-cultural relevance of beneficial parenting practices, such programs may be adapted to accommodate diverse cultural norms and values, including collectivist and individualist orientations. Tailoring intervention content to cultural expectations surrounding parental roles and family organization may further enhance effectiveness.

At the policy level, integrating comprehensive parental education programs into existing educational and community frameworks may offer a scalable avenue for support. Programs aligned with children’s developmental stages and key family transition points, such as entry into preschool or primary school, could provide timely and developmentally appropriate guidance. A structured, ongoing model that combines initial training with periodic refresher sessions may help parents sustain effective parenting practices over time, ultimately supporting both child development and parental well-being.

### 4.5. Limitations and Future Directions

Several limitations should be acknowledged. Participants were recruited via snowball sampling, and all data were collected via self-report measures, which may be subject to social desirability bias. In addition, the predominance of middle-class families in the sample, along with the introduction of the general well-being questionnaire after the pilot phase, which made it available only to a subset of participants, may limit generalizability and reduce statistical precision for analyses involving general well-being. Accordingly, the magnitude of the observed associations and asymmetric cross-parent patterns should be interpreted with caution, as shared method variance, sample characteristics, and measurement timing may have influenced the strength and stability of the reported relationships.

A further limitation concerns the cross-sectional design of the study, which precludes conclusions about temporal ordering or directionality of effects. Although the APIM framework allows for the simultaneous estimation of actor and partner associations, longitudinal designs are needed to determine how parenting behaviors and parents’ general and parental well-being influence one another over time. In particular, future research should examine whether the observed asymmetry in cross-parent associations reflects stable relational dynamics or developmental stage–specific processes.

Future studies employing longitudinal and observational methods would also help reduce reliance on shared self-report data and strengthen inference regarding dyadic processes. Research using more representative sampling strategies and incorporating reports from external informants (e.g., observational coding or partner reports) would further enhance validity. Finally, because the present study focused on parents of young children in Israel, cross-cultural replication is needed to assess the generalizability of the findings to other populations, cultural contexts, and family structures.

## Figures and Tables

**Table 1:** Description of parents’ report of their parenting behaviors (PPM) and their well-being, along with comparisons between mothers and fathers (*N* = 85).

	Mothers		Fathers			
	Range	*M* (*SD*)	Range	*M* (*SD*)	*t*	Within-Couple *r*
The PPM						
Partnership	1.43–5.79	4.48 (0.83)	3.23–5.86	4.58 (0.66)	−1.43	0.65 ***
Leadership	3.11–5.67	4.54 (0.55)	3.22–5.78	4.52 (0.51)	0.36	0.54 ***
Expressions of Love	2.82–5.88	4.86 (0.57)	3.06–5.71	4.70 (0.57)	2.38 **	0.44 ***
Encouraging Independence	3.36–5.55	4.28 (0.48)	2.45–5.27	4.23 (0.53)	0.80	0.27 **
Adherence to Rules	2.43–5.50	4.27 (0.60)	3.36–5.64	4.30 (0.52)	−0.29	0.49 ***
General well-being						
Positive feelings	2.36–5.91	4.15 (0.83)	2.36–5.55	4.24 (0.68)	−0.80	0.38 **
Negative feelings	1.33–4.80	2.82 (0.76)	1.50–3.89	2.48 (0.61)	3.07 **	0.21
Parental well-being						
Positive feelings	2.90–6.00	4.92 (0.79)	3.00–6.00	4.88 (0.70)	0.38	0.41 ***
Negative feelings	1.00–5.09	2.74 (0.82)	1.00–4.45	2.51 (0.71)	2.32 *	0.31 **

*Note.* Values are means with standard deviations in parentheses. *t* values are from paired-samples *t* tests comparing mothers and fathers. *r* represents within-couple (mother–father) Pearson correlations. Possible scale range = 1–6. General well-being data were available for 65 mothers and 65 fathers. * *p* < 0.05; ** *p* < 0.01; *** *p* < 0.001.

**Table 2:** General Well-Being as a Function of Five Parenting Constructs (*N* = 65).

	Mother					Father				
	**Number of Children**	**Actor**	**Partner**	**IV Mother with Father**	***R* ^2^**	**Number of Children**	**Partner**	**Actor**	**DV Mother with Father**	***R* ^2^**
Partnership	0.08 (0.08)	0.72 *** (0.10)	0.04 (0.11)	0.68 *** (0.07)	0.56 *** (0.08)	−0.14 (0.11)	0.09 (0.14)	0.46 ** (0.14)	−0.04 (0.12)	0.30 ** (0.09)
Leadership	0.11 (0.09)	0.63 *** (0.10)	0.04 (0.10)	0.60 *** (0.08)	0.43 *** (0.07)	−0.14 (0.10)	0.09 (0.14)	0.46 ** (0.13)	0.10 (0.12)	0.21 * (0.02)
Expressions of Love	0.17 (0.10)	0.62 *** (0.10)	−0.06 (0.12)	0.56 *** (0.09)	0.38 *** (0.10)	−0.11 (0.11)	−0.02 (0.13)	0.46 *** (0.12)	0.24 * (0.12)	0.22 * (0.09)
Encouraging Independence	-	0.23 * (0.09)	0.44 *** (0.09)	0.30 ** (0.11)	0.30 *** (0.06)	-	−0.06 (0.13)	0.25 * (0.12)	0.23 * (0.12)	0.06 (0.05)
Adherence to Rules	0.13 (0.09)	0.40 *** (0.11)	0.21 * (0.10)	0.51 *** (0.09)	0.31 *** (0.06)	Set to 0	0.15 (0.13)	0.26 * (0.13)	0.20 (0.12)	0.13 (0.08)
**Model Goodness-of-Fit**	**Chi-Square**	***df***	***p***	**CFI**	**TLI**	**RMSEA**	**SRMR**			
Partnership	0.29	2	0.87	1.00	1.00	0.00	0.015			
Leadership	1.67	3	0.65	1.00	1.00	0.00	0.048			
Expressions of Love	2.73	2	0.25	0.982	0.937	0.075	0.065			
Encouraging Independence	0.91	1	0.34	1.00	1.00	0.00	0.092			
Adherence to Rules	4.73	4	0.32	0.960	0.931	0.053	0.150			

*Note*. To achieve satisfactory model goodness-of-fit, the number of children variable was excluded from the Independence model (indicated by a dash), and non-significant paths were constrained to zero (indicated by “Set to 0”). Standard errors of the estimates are in parentheses; * *p* < 0.05. ** *p* < 0.01. *** *p* < 0.001. Actor effect = the association between a parent’s own parenting construct and their own general well-being. Partner effect = the association between one parent’s parenting construct and the partner’s general well-being.

**Table 3:** Parental well-being as a function of five parenting constructs (*N* = 85).

	Mother					Father				
	**Number of Children**	**Actor**	**Partner**	**IV Mother with Father**	***R* ^2^**	**Number of Children**	**Partner**	**Actor**	**DV Mother with Father**	***R* ^2^**
Partnership	-	0.66 *** (0.09)	0.003 (0.11)	0.66 *** (0.06)	0.44 *** (0.08)	-	0.13 (0.12)	0.50 *** (0.11)	Set to 0	0.35 *** (0.08)
Leadership	−0.17 ** (0.06)	0.64 *** (0.07)	0.17 * (0.08)	0.54 *** (0.08)	0.58 *** (0.06)	−0.15 (0.08)	−0.04 (0.09)	0.70 *** (0.08)	0.02 (0.11)	0.48 *** (0.08)
Expressions of Love	−0.10 (0.06)	0.73 *** (0.06)	0.05 (0.08)	0.44 *** (0.09)	0.57 *** (0.06)	−0.15 * (0.08)	−0.04 (0.08)	0.74 *** (0.06)	0.19 (0.11)	0.55 *** (0.07)
Encouraging Independence	−0.33 *** (0.09)	0.29 ** (0.09)	0.26 ** (0.09)	0.29 ** (0.10)	0.31 *** (0.08)	−0.16 (0.10)	0.02 (0.11)	0.35 *** (0.10)	0.23 * (0.10)	0.15 * (0.07)
Adherence to Rules	−0.31 ** (0.09)	0.44 *** (0.10)	0.02 (0.11)	0.49 *** (0.08)	0.29 *** (0.08)	−0.13 (0.10)	0.10 (0.11)	0.32 ** (0.11)	0.25 * (0.10)	0.16 * (0.07)
**Model Goodness-of-Fit**	**Chi-Square**	***df***	***p***	**CFI**	**TLI**	**RMSEA**	**SRMR**			
Partnership	0.58	1	0.45	1.00	1.00	0.00	0.013			
Leadership	3.02	3	0.39	1.00	1.00	0.009	0.051			
Love	7.04	3	0.07	0.970	0.931	0.126	0.078			
Encouraging Independence	2.11	2	0.35	0.997	0.991	0.025	0.046			
Adherence to Rules	1.38	2	0.50	1.00	1.00	0.00	0.026			

*Note*. Standard errors are shown in parentheses. To achieve a satisfactory model fit, the number of children variable was excluded from the partnership model (indicated by a dash), and nonsignificant paths were constrained to zero (indicated by “set to 0”). * *p* < 0.05. ** *p* < 0.01. *** *p* < 0.001.

## Data Availability

The data presented in this study are available from the corresponding author upon reasonable request. The Parenting Pentagon Model (PPM) questionnaire has been independently published and applied in prior peer-reviewed journal articles and book chapters, and the full set of PPM items is available from the corresponding author upon reasonable request for research or clinical use.

## Appendix A

**Table A1:** Correlations Between PPM Behaviors and Mothers’ and Fathers’ General/Parental Well-Being.

	Mother		Fathers	
PPM Behavior	General Well-Being	Parental Well-Being	General Well-Being	Parental Well-Being
**Mothers**				
Partnership	0.74 **	0.67 **	0.41 **	0.46 **
Leadership	0.66 **	0.73 **	0.32 **	0.36 **
Expressions of Love	0.57 **	0.75 **	0.26 *	0.32 **
Encouraging Independence	0.29 *	0.32 **	−0.01	0.10
Adherence to Rules	0.44 **	0.43 **	0.26 *	0.25 *
**Fathers**				
Partnership	0.53 **	0.44 **	0.52 **	0.59 **
Leadership	0.45 **	0.48 **	0.45 **	0.68 **
Expressions of Love	0.28 *	0.36 **	0.46 **	0.73 **
Encouraging Independence	0.45 **	0.34 **	0.21	0.35 **
Adherence to Rules	0.30 *	0.25 *	0.31 *	0.38 **

*Note*. Values are Pearson correlation coefficients. *p* < 0.05. *p* < 0.01 (two-tailed). Parental well-being correlations based on *N* = 85 dyads; General well-being correlations based on *N* = 65 dyads. * *p* < 0.05. ** *p* < 0.01.
